# Genetic and Epigenetic Modifications of Sox2 Contribute to the Invasive Phenotype of Malignant Gliomas

**DOI:** 10.1371/journal.pone.0026740

**Published:** 2011-11-01

**Authors:** Marta M. Alonso, Ricardo Diez-Valle, Lorea Manterola, Angel Rubio, Dan Liu, Nahir Cortes-Santiago, Leire Urquiza, Patricia Jauregi, Adolfo Lopez de Munain, Nicolás Sampron, Ander Aramburu, Sonia Tejada-Solís, Carmen Vicente, María D. Odero, Eva Bandrés, Jesús García-Foncillas, Miguel A. Idoate, Frederick F. Lang, Juan Fueyo, Candelaria Gomez-Manzano

**Affiliations:** 1 Department of Neuro-oncology, The University of Texas MD Anderson Cancer Center, Houston, Texas, United States of America; 2 Department of Oncology, University Hospital of Navarra, Pamplona, Spain; 3 Department of Neurosurgery, University Hospital of Navarra, Pamplona, Spain; 4 Division of Oncology, BioDonostia Institute, San Sebastian, Spain; 5 Department of Biostatistics, Centro de Estudios e Investigaciones Técnicas de Guipuzcoa (CEIT), San Sebastian, Spain; 6 Division of Oncology, Center for Applied Medical Research, University of Navarra, Pamplona, Spain; 7 Department of Pathology, University Hospital of Navarra, Pamplona, Spain; 8 Department of Neurosurgery, The University of Texas MD Anderson Cancer Center, Houston, Texas, United States of America; The University of Arizona, United States of America

## Abstract

We undertook this study to understand how the transcription factor Sox2 contributes to the malignant phenotype of glioblastoma multiforme (GBM), the most aggressive primary brain tumor. We initially looked for unbalanced genomic rearrangements in the Sox2 locus in 42 GBM samples and found that Sox2 was amplified in 11.5% and overexpressed in all the samples. These results prompted us to further investigate the mechanisms involved in Sox2 overexpression in GBM. We analyzed the methylation status of the Sox2 promoter because high CpG density promoters are associated with key developmental genes. The Sox2 promoter presented a CpG island that was hypomethylated in all the patient samples when compared to normal cell lines. Treatment of Sox2-negative glioma cell lines with 5-azacitidine resulted in the re-expression of Sox2 and in a change in the methylation status of the Sox2 promoter. We further confirmed these results by analyzing data from GBM cases generated by The Cancer Genome Atlas project. We observed Sox2 overexpression (86%; N = 414), Sox2 gene amplification (8.5%; N = 492), and Sox 2 promoter hypomethylation (100%; N = 258), suggesting the relevance of this factor in the malignant phenotype of GBMs. To further explore the role of Sox2, we performed in vitro analysis with brain tumor stem cells (BTSCs) and established glioma cell lines. Downmodulation of Sox2 in BTSCs resulted in the loss of their self-renewal properties. Surprisingly, ectopic expression of Sox2 in established glioma cells was not sufficient to support self-renewal, suggesting that additional factors are required. Furthermore, we observed that ectopic Sox2 expression was sufficient to induce invasion and migration of glioma cells, and knockdown experiments demonstrated that Sox2 was essential for maintaining these properties. Altogether, our data underscore the importance of a pleiotropic role of Sox2 and suggest that it could be used as a therapeutic target in GBM.

## Introduction

Therapy for malignant gliomas is currently suboptimal. A better understanding of the cellular and molecular features of these tumors should propel the development of more effective therapies. In 2004, two independent groups identified brain tumor stem cells (BTSCs) as the tumor-initiating cells of glioblastoma multiforme (GBM) [1], [2]. This population of cells is thought to be responsible for the invariable recurrence of GBM after treatment. BTSCs possess traits similar to those of neural stem cells, such as the property of self-renewal and the expression of several embryonic stem cell markers. Moreover, a link has been reported between aberrant expression levels of these embryonic stem cell markers and the histological grade of gliomas [3], [4]. Thus, abnormalities in genes and pathways involved in the regulation of stem cell self-renewal seem to be important in the stem cell model of cancer. In agreement with this notion, Sox2, a critical transcription regulator of embryonic and neural normal stem cell function [3], [4], has been reported to be dysregulated in several human cancers. Sox2 was found to be frequently downregulated in gastric cancers [5] and overexpressed in small-cell lung cancers, esophageal squamous carcinomas, and basal cell-like breast carcinomas [3], [6], [7]. In addition, the Sox2 locus was shown to be amplified in small-cell lung cancer, esophageal squamous carcinomas [6], [7], and, to a lesser extent, in GBM [8].

Although overexpression of Sox2 mRNA has been reported in malignant gliomas when compared with nonmalignant tissue [4], an exhaustive molecular and mechanistic analysis of Sox2 has never been performed. Therefore, the main purpose of this study was to further investigate the status of Sox2 in GBM at the molecular level and to elucidate how this transcription factor contributes to the malignant phenotype of this lethal disease.

## Results and Discussion

**Figure 1:**
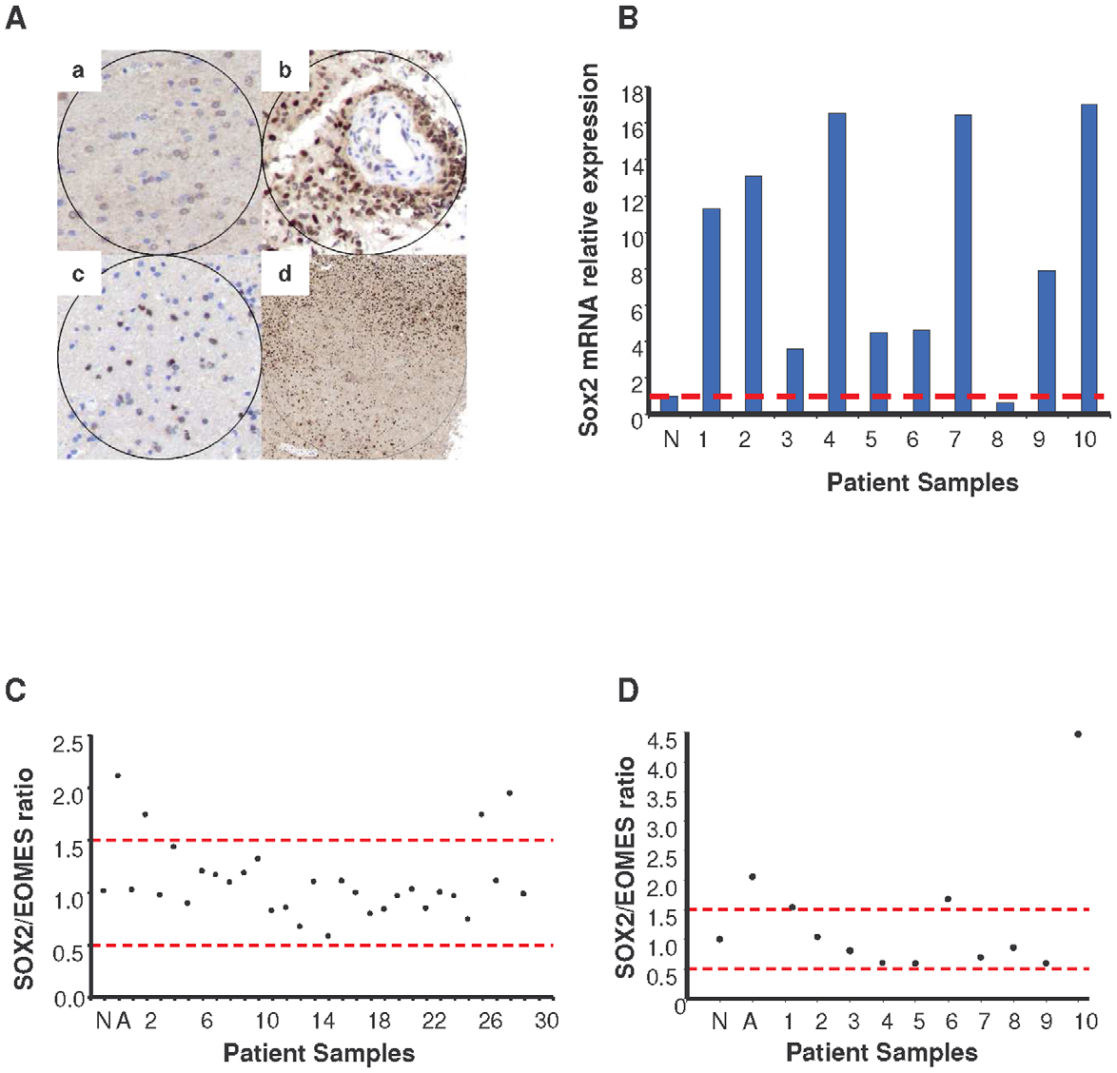
Molecular analysis of Sox2 in GBM specimens. **A.** Sox2 was overexpressed at the protein level in GBM samples. Representative micrographs of Sox2 immunostaining in GBM samples (n = 30). **a**, normal white matter with slight cytoplasmic staining in the cytoplasm; **b**, GBM with intense nuclear staining in tumor cells and no vascular staining; **c**, Sox2-positive infiltrating cells in normal tissue around the tumor (20×); **d**, infiltrative border with Sox2-positive tumor cells (4×). **B.** Sox2 was overexpressed at the mRNA level in GBM samples. This panel illustrates the relative Sox2 mRNA expression levels in 10 GBM samples. The red dashed line represents the normal threshold. N = normal brain. **C and D.** Amplification of Sox2 in the two independent cohorts (left, 30 GBM samples; right, 10 GBM samples). N = normal human astrocytes; A = leukemia cell line TF1 (amplified Sox2). The red dashed lines represent the normal threshold.

First, we investigated Sox2 expression in two independent cohorts of 30 and 10 human surgical GBM specimens, respectively. We found that Sox2 was overexpressed at the protein level in all the analyzed tumors in the first cohort (30/30), and screening revealed mRNA overexpression in 90% of the specimens in the second cohort (9/10) (Figure 1A and 1B). Interestingly, Sox2-positive cells were frequently observed in the invasive tumoral front, but endothelial and inflammatory cells did not stain positive for Sox2 (Figure 1A; and Figure S1A; and data not shown). Since overexpression could be due to unbalanced genomic rearrangements in the Sox2 locus, we analyzed the genetic information of these tumors to determine Sox2 amplification and found that Sox2 was amplified in 3/30 (10% incidence) and 3/10 (30% incidence) of these samples (Figure 1C and 1D).

**Figure 2:**
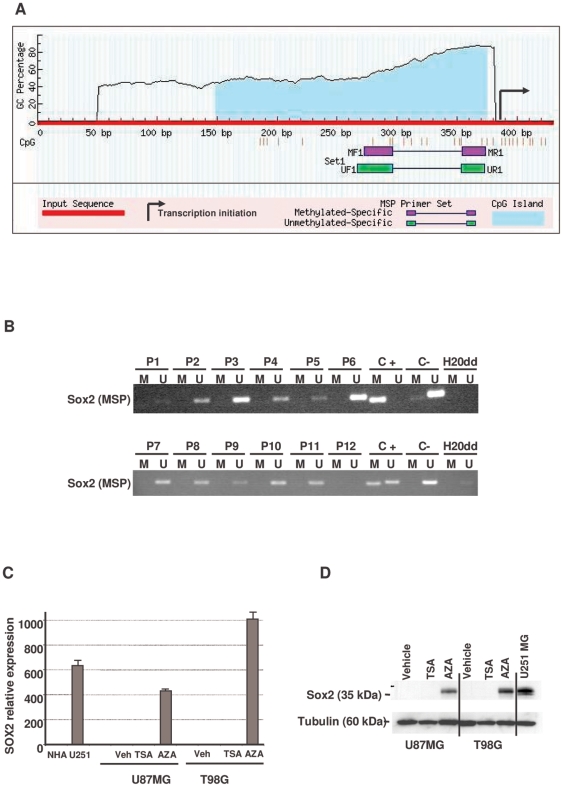
Methylation analysis of the *Sox2* promoter gene. **A.** Analysis of the *Sox2* promoter CpG content. The position of the *Sox2* promoter CpG island is shown. The vertical tic marks denote the CpGs in the island, and the position of the DNA methylation assay with MSP primers is shown. B. MSP analysis of the promoter CpG islands of *Sox2* in 12 patients with GBM. PCR products recognizing unmethylated (U) and methylated (M) CpG sites were analyzed on 2% agarose gels. C+ = positive control; in vitro methylated control, C− = negative control; DNA from normal brain and ddH_2_O = water control containing no DNA. **C.** Relative expression of SOX2 mRNA following treatment with either vehicle (Veh), Trichostatin A (TSA), or 5-aza-2′-deoxycytidine (AZA;). Data were averaged from three independent experiments. Normal human astrocytes (NHAs) and U251 MG cell line were used as negative and positive controls, respectively. **D.** Analysis of the expression of Sox2 protein following the same treatment described in **C.** Data were averaged from three independent experiments. The U251 MG cell line was used as positive control.

Next, we analyzed Sox2 expression in BTSCs and established glioma cell lines. Sox2 mRNA expression was significantly higher in BTSCs than in the established glioma cell lines (Figure S1B), and Sox2 protein was more frequently detected in BTSCs (6/7) than in established glioma cell lines (4/8) (Figure S1C). Sox2 amplification was present in both BTSCs (2/7) and established glioma cell lines (1/7) (Figure S1D). These results prompted us to further investigate the mechanisms involved in Sox2 overexpression in GBM. We first analyzed the methylation status of the Sox2 promoter because high-CpG-density promoters are associated with key developmental genes [9]. Our data revealed that the Sox2 promoter presented an extensive CpG island before the transcription start site (Figure 2A) that included part of the coding region (Figure S2A). Analysis using methylation-specific polymerase chain reaction ([PCR] MSP) of 12 GBM samples showed that the Sox2 promoter was hypomethylated in all 12 samples when compared to normal human astrocytes (Figure 2B and S2B). Assessment of Sox2 promoter methylation status in BTSCs and established glioma cell lines confirmed that the Sox2 promoter was hypomethylated in Sox2-expressing cell lines. In contrast, Sox2-negative cell lines exhibited a methylated promoter pattern (Figure S1B and S2B). Interestingly, this hypomethylated pattern in the Sox2 promoter mirrors the one found in neural precursor cells [10], [11], [12], suggesting that GBMs and BTSCs display a more undifferentiated phenotype. To rule out other possible epigenetic regulatory mechanisms, we treated two Sox2-negative cell lines (U-87 MG and T98G) with the histone deacetylase inhibitor trichostatin A (TSA) or with the DNA methyl-transferase inhibitor 5-azacitidine (AZA). Treatment with AZA, but not TSA, resulted in the re-expression of Sox2 at the mRNA and protein levels (Figure 2C and 2D), further supporting a pivotal role of aberrant DNA promoter demethylation in the regulation of Sox2 levels in GBM.

**Figure 3:**
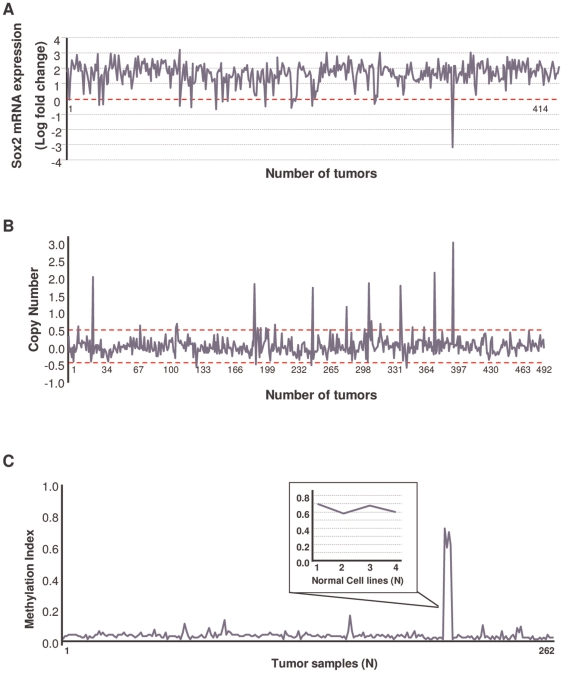
TCGA Data Meta-analysis. **A.** Relative expression of Sox2 mRNA in 414 GBM samples from TCGA. Sox2 was overexpressed in 86% of the samples (357/414). The red dashed lines represent the normal threshold. **B.** Amplification of the Sox2 locus in 492 GBM specimens from TCGA. Sox2 showed amplification in the number of copies (copy number ≥2.5 copies) in 8.5% of the samples (42/492). The red dashed lines represent the normal copy number. **C.** Sox2 methylation status of 262 samples (258 tumors and 4 normal cell lines). These data show that Sox2 is methylated in the normal cell lines and hypomethylated in 100% of the tumor samples.

We validated these results by analyzing data from The Cancer Genome Atlas (TCGA; [13], available at https://cgwb.nci.nih.gov/cgi-bin/heatmap#TCGAGBM). In the sets of GBM specimens from TCGA, we found Sox2 mRNA overexpression in 86.2% of the samples (357/414) (Figure 3A), amplification of the Sox2 locus (3q26.33) copy number in 8.5% of the samples (42/492) (Figure 3B), and hypomethylation of the promoter in 100% of the samples when compared with normal controls (N = 258) (Figure 3C), again suggesting the relevance of this transcription factor in the malignant phenotype of GBMs. Together, our results indicate that Sox2 overexpression in GBM is regulated at the genetic and epigenetic levels and that DNA promoter demethylation is the leading cause of Sox2 deregulation.

**Figure 4:**
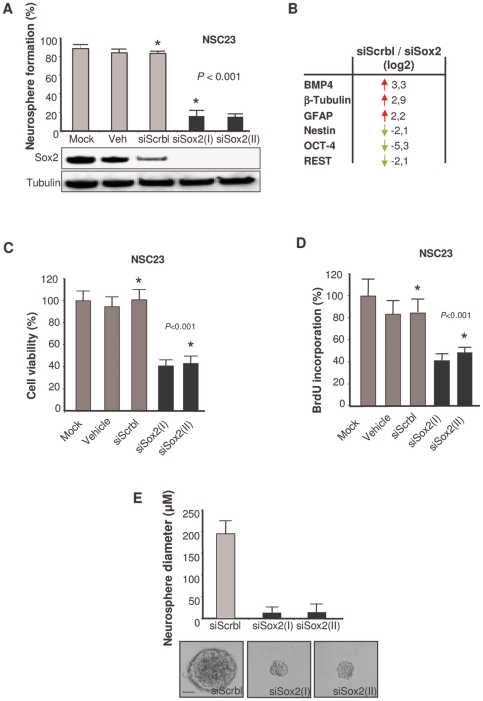
Role of Sox2 in self-renewal properties of GBM. **A.** Role of Sox2 in the self-renewal capabilities of BTSCs. Upper panel, quantification of neurosphere formation percentage in Sox2-silenced NSC23 BTSC line. NSC23 cells were transfected with mock, vehicle (Veh), siRNA scramble (siScrbl), or two different siRNAs against Sox2, Sox2 (I) and Sox2 (II), and the number of secondary spheres generated was assessed after 8–10 days. To confirm that the spheroids were, indeed, formed by stem cells, we randomly selected at least 15 individual secondary spheres and subjected them to further, long-term (2 months) propagation in each subcloning experiment. Lower panel, representative western blot of Sox2-silenced NSC23 cell line. **B.** Assessment of differentiation/stem cell markers in Sox2-silenced BTSCs. The expression values of the markers were expressed as the log2 ratio of the Sox2-silenced sample (siSox2) versus the siScramble-transfected sample (siScrbl). Data shown are from one representative experiment. **C.** Cell viability analysis in Sox2-silenced BTSCs. Cell viability was examined 7 days later by MTT assay. **D.** Cell proliferation analysis in Sox2-silenced BTSCs. Cell proliferation was evaluated by measuring the BrdU incorporation and expressed as a percentage of the mock-treated cells. **E.** Neurosphere size evaluation in Sox2-silenced BTSCs. Upper panel, Quantification of the diameter of NSC23 neurosphere transfected with siScrbl or two different siRNAs against Sox2, Sox2(I) and Sox2(II). Lower panel, representative micrographs of neurosphere size are depicted (50 µm magnification).

**Figure 5:**
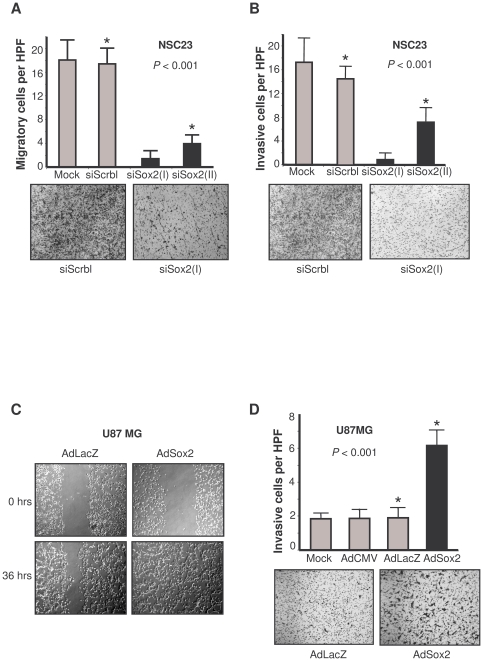
Role of Sox2 in the migration and invasion properties of GBM. **A.** Assessment of cell migration of Sox2-silenced BTSCs. Sox2 regulates positively the migration ability of BTSCs. For transwell migration assays, NSC23 cells were untreated or transfected with siRNA scramble, siRNA Sox2(I), or siRNA Sox2(II). Quantification of the migration is expressed as the number of migrating cells per high-power field (HPF; ×40). Bottom, representative micrographs of the transwell migration assay (10× magnification). **B.** Assessment of cell invasion in Sox2-silenced BTSCs by transwell invasion. Sox2 regulates positively the invasion ability of BTSCs. For transwell invasion assays, NSC23 cells were left untreated or transfected with siRNA scramble, siRNA Sox2(I), or siRNA Sox2(II). Quantification of the invasion is expressed as the number of invasive cells per HPF. Bottom, representative micrographs of the transwell invasion assay (10× magnification). **C.** Assessment of migration in Sox2-overexpressing glioma cell lines by wound assay. U-87 MG cells were left untreated or infected with 100 multiplicities of infection (MOIs) of an adenovirus vector containing an empty cassette CMV (AdCMV), an adenovirus carrying LacZ cDNA (AdLacZ), or an adenovirus carrying Sox2 cDNA (AdSox2). Twenty-four hours later, 1×10^5^ cells were seeded in six-well plates and left until they reached 80% confluency. At that time (0 h), we performed a scratch with a pippette tip, and repopulation of the wound was observed by light microscopy. **D.** Assessment of invasion in Sox2-overexpressing glioma cell lines by transwell invasion. U-87 MG cells were left untreated or infected with 100 MOIs of an AdCMV, an AdLacZ, or an AdSox2. Invasion using Matrigel-coated transwell was performed as in **B.** Quantification of the invasion is expressed as the number of invasive cells per HPF. Bottom, representative micrographs of the transwell invasion assay (10× magnification).

Next, to understand the functional role of Sox2 in GBM, we knocked down Sox2 expression in NSC23 BTSCs by using two independent small interfering RNAs (siRNAs). Downmodulation of Sox2 jeopardized the neurosphere-forming activity, as indicated by the results of both clonal neurosphere (Figure 4A) and limited dilution assays (Figure S3A), and drove the cells to display a differentiation expression profile (Figure 4B), thus indicating impairment of the self-renewal phenotype of BTSCs. Moreover, suppression of Sox2 prompted a decrease in cell viability (Figure 4C), a decrease in bromodeoxyuridine (BrdU) incorporation (Figure 4D), accumulation of cells in the G0/G1 cell cycle phase (Figure S3B), and a decrease in proliferation markers such as E2F1 (Figure S3C). These phenotypic modifications were evidenced by the generation of smaller neurospheres from Sox2-knockdown cells than from parental BTSCs (Figure 4E). These data indicate Sox2 is involved in the maintenance of a more undifferentiated phenotype that includes the capacity of self-renewal. We further confirmed these data in another BTSC line, NSC11 (Figure S4A–S4D). Strikingly, when we introduced Sox2 in the Sox2-negative established cell line U-87 MG, we did not observe the acquisition of self-renewal or neurosphere-forming properties (Figure S5A–S5D), although we detected a modest but significant increase in cell viability and proliferation (Figure S5E and S5F). Similarly, established glioma cultures with endogenous Sox2 overexpression did not display neurosphere formation when cultured in enriched growth factor medium (Figure 5G). Collectively, our data indicate that Sox2 is essential but not sufficient to sustain self-renewal and that other factors cooperate to activate stem cell-like properties in glioma cells.

Because infiltration and invasion are the main characteristics of the malignant phenotype of gliomas, we sought to ascertain whether Sox2 plays a role in the invasion/migration potential of malignant gliomas. Downregulation of Sox2 using siRNA resulted in a significant decrease in the migratory (Figure 5A) and invasive capabilities (Figure 5B) of not only BTSCs (Figure S6A) but also established glioma cells that express Sox2 (Figure S6A) This phenomenon was associated with a significant decrease in the expression of several proteins involved in migration, invasion, and angiogenesis (Figure S6B). Interestingly, restoration of Sox2 expression in a Sox2-negative glioma cell line, U-87 MG, resulted in a significant increase in the percentage of migratory and invasive cells (Figure 5C, 5D and S6C).

Here, we present the first comprehensive report, to our knowledge, of Sox2 overexpression mechanisms in GBM and reveal a previously undescribed role of this transcription factor in the invasion and migration properties of glioma BTSCs and cell lines. We identified Sox2 promoter DNA hypomethylation as the leading mechanism responsible for Sox2 aberrant expression in GBM. The fact that the percentage of Sox2 amplification in GBMs (13%) did not justify the high frequency of Sox2 overexpression in these tumors prompted us to search for other molecular mechanisms, such as epigenetic dysregulation of the Sox2 promoter or its regulators. In this regard, DNA hypomethylation and histone acetylation are two of the mechanisms direct the pluripotency/differentiation fate of neural stem cells [11], [14]. Our data revealed that Sox2 presents a high CpG density throughout the promoter. These data are in accordance with other studies that found that the Sox2 locus is flanked by two bivalent CpG islands that, under certain physiological conditions, may poise the gene for repression upon differentiation [12]. In this regard, our results suggest that the Sox2 promoter hypomethylation we observed in GBM reflects a more primitive cellular state resembling that found in neural stem cells [12]. Even though our findings suggest that DNA promoter hypomethylation is the major contributing factor to Sox2 overexpression, others have shown that DNA methylation patterns can be better explained by histone methylation patterns than by CpG density [12], [15]. Interestingly, in embryonic and neural stem cells, the Sox2 promoter is enriched with the transcription initiation mark H3k4me3, and this mark resolves to H3k27me3 (which is known to be repressive) in differentiated cells [12], [16]. Therefore, we could not rule out that in GBM, the Sox2 promoter presents an enrichment of the positive transcription mark H3k4me3; further experiments are needed to test this hypothesis.

According to data from the present study and others [17], Sox2 overexpression in BTSCs is essential for maintaining the self-renewal of BTSCs, as assessed by neurosphere-forming activity and expression levels of pluripotent markers. Importantly, data from our study suggest the involvement of critical factors besides Sox2 in maintaining self-renewal. This function mimics a well-described role of Sox2 in the developing central neural system, in which Sox2 plays a critical role in maintaining the self-renewal and pluripotency of the neural stem cells [18] in a coordinated fashion with other transcriptional factors, such as Oct4 and Klf4 or c-Myc [19], [20].

Sox2 has been shown to maintain neural stem cell identity in the developing brain. Constitutive expression of Sox2 inhibited neuronal differentiation and preserved neural progenitor characteristics, whereas inhibition of Sox2 led to an early onset of neuronal differentiation [21], [22]. In the adult brain, Sox2 expression is retained in the neural stem cell compartment [23]. Interestingly, reduced expression of Sox2 in adult mice resulted in a substantial reduction in proliferating neural precursors, suggesting an important role of Sox2 in neural stem cell maintenance or proliferation in the adult brain [23], [24]. In addition, our data show that Sox2-driven malignant glioma cells are highly invasive and have migratory characteristics mimicking those of neural stem cells [25]. This finding supports those of other studies which showed that Sox2 is expressed in the rostral migratory stream [23] and in the migratory cells of the developing pituitary gland [26].

In our model, Sox2 was sufficient and essential for maintaining the invasive properties of glioma cell lines and BTSCs. However, further analysis is needed to address the in vivo role of Sox2 in the migration and invasion in GBM. On the basis of our data, we propose that Sox2 acts as an oncogene in GBM and that its reactivation in glioma cells, due to its genomic amplification and promoter hypomethylation, could be responsible for the acquisition of the invasive phenotype. Elucidation of the different Sox2-related pathways that separate normal stem cells from BTSCs should guide us in designing new and safe therapies for patients with GBM.

## Materials and Methods

### Cell Lines and Culture Conditions

The glioma cell lines LN229, U87 MG, U251 MG, U373, A172, SNB19, D54, and T98G and the cell line NTERA2 were obtained from the American Type Culture Collection. Cell lines were maintained in Dulbecco's modified Eagle's medium/nutrient mixture F12 (1∶1, vol/vol) supplemented with 10% fetal bovine serum in a humidified atmosphere containing 5% CO_2_ at 37°C. Normal human astrocytes (NHAs) were purchased from Clonetics/BioWhittaker (Walkersville, MD) and were maintained in astrocyte growth medium (AGM; in an AGM BulletKit) obtained from Clonetics/BioWhittaker. Neurosphere cultures were established from acute cell dissociation of human GBM surgical specimens [1], [27] and maintained in Dulbecco's modified Eagle's medium/nutrient mixture F12 supplemented with B27 (Invitrogen, Carlsbad, CA), epidermal growth factor, and basic fibroblast growth factor (20 ng/mL each; Sigma-Aldrich, St Louis, MO).

### Sox2 Expression Levels by Quantitative PCR

The expression levels of Sox2 transcripts were quantified relative to the expression level of the housekeeping gene glyceraldehyde-3-phosphate dehydrogenase (GAPDH) by real-time PCR (RT-PCR) using an ABI 7700 sequence detection system (Applied Biosystems, Foster City, CA). The expression levels relative to GAPDH were calculated using the ddCt method and normalized with normal brain RNA (N) [28]. Primer sequences are included in Text S1.

### Assessment of SOX2 Amplification

To assess Sox2 amplification, we used the comparative ddCt method with SYBR-green (LightCycler 480 SYBR Green I Master; Roche, Manheim, Germany) for quantitative PCR (Q-PCR). Primers were designed with PrimerExpress software (Applied Biosystems), and we validated whether the efficiency of the amplification of the chosen primer sets was equal to that of the normalizer. A primer set for the EOMES and SLITRK3 genes was used for normalization. We used the acute leukemia cell line TF1 as a positive control for amplification (A) and NHAs as a negative control (NHA). The validation experiments were performed on fourfold serial dilutions of genomic DNA, starting with 100 ng in the first dilution. For relative quantification, the reaction mixtures consisted of LightCycler 480 SYBR Green I Master (Roche) with 500 nM of each primer and 10 ng DNA in a total volume of 25 µl. After an initial denaturation step for 10 min at 95°C, thermal cycling conditions were 15 s at 95°C and 1 min at 60°C for 40 cycles. Finally, the dissociation curves for each reaction were determined. All samples were run in duplicate or triplicate on an ABI 7700 sequence detection system (Applied Biosystems). Primer sequences are included in Text S1.

### Primer Design MSP Analysis

Primer sequences for MSP analysis were designed using MSPPrimer [29], and their location in the SOX2 promoter is indicated in Figure 2A. All primer sequences are included in Text S1.

### Assessment of SOX2 Promoter Methylation

For MSP analysis, DNA was extracted from cell lines and patients using the DNeasy Kit (Quiagen; Valencia, CA). Bisulfite modification of genomic DNA was carried out using the EZ DNA methylation kit (Zymo Research; Irvine, CA). We analyzed SOX2 promoter methylation using MSP primer pairs covering the putative transcriptional start site in the 5′CpG island with 1 µL of bisulfite-treated DNA as a template and JumpStart Red Taq DNA polymerase (Sigma) for amplification, as previously described [30].

### Demethylation Studies

For demethylation studies, cultured cells were treated with 1 µmol/L 5-aza-2′-deoxycytidine (Sigma) for 72 h, with the medium changed every 24 h. Trichostatin A (Sigma) was used to treat cells at a concentration of 300 nmol/L for 18 h. Vehicle drug treatments were performed in parallel with drug-free media.

### TCGA Data Meta-analysis

Results of copy number and gene expression status were downloaded from the TCGA data portal. All the copy number data from samples hybridized by Memorial-Sloan Kettering Cancer Center using the Agilent Human Genome CGF microarray 244A (HG-CGH-244A) were downloaded. This dataset included 492 matched pairs of tumor and normal samples. The pipeline of TCGA directly provided the estimated copy number for each of the probes in the array (summarization level 2, according to TCGA). In particular, SOX2 was interrogated by three different probes. The provided copy number is the average of the copy numbers predicted by each probe. SOX2 was amplified in the predicted copy numbers (copy number ≥2.5 copies) in 8.5% of the samples (42/492).

To determine gene expression, the analyzed selected samples were hybridized by The Broad Institute using an Affymetrix HG-U133A array (Affymetrix; Santa Clara, CA). It included 414 tumor samples plus 11 normal samples (not matched with the previous ones). Two probe sets (213721_at and 213722_at) hit the SOX2 gene. The average “normal” expression of the SOX2 gene for each probe set was computed by taking the mean of the logarithms of the signals of the probesets for normal samples. Once this reference was obtained, the logarithm of the fold change for each of the samples was computed by substracting this reference value from the log of the signal of each of the probes. The displayed fold change was the average fold change for both probe sets. Concordance between both probe sets was very strong (Pearson correlation coefficient = 0.87, p≪1e-16). SOX2 was overexpressed at least twofold in 86% of the samples (357/414). Finally, the selected data that were used to estimate the methylation status were hybridized by the Johns Hopkins University group using the Illumina GoldenGate Methylation Cancer Panel I. It included the methylation status of 262 samples (258 tumors and normal cell line in quadruplicate; B lymphocyte cat# GM06990, Coriell Institute for Medical Research, Camden, NJ). These data show that SOX2 was methylated in the normal cell lines and hypomethylated in 100% of the tumor samples.

### Self-renewal Analysis

Cells derived from the dissociation of clonal single neurospheres were seeded in 96-well plates, and the number of generated secondary spheres was assessed after 8–10 days. To avoid including the colonies that may have been formed by transient amplification of cells in these cultures, we counted only secondary spheres that exceeded 120 µm in diameter. To confirm that the latter were, indeed, formed by stem cells, we randomly selected at least 15 individual secondary spheres and subjected them to further, long-term (2 months) propagation in each subcloning experiment.

### Immunoblotting Assays

For the immunoblotting assays, cells were lysed in (3-[(3-cholamidopropyl)-dimethylammonio]-1-propane sulfonate) buffer for 30 min on ice. Samples containing identical amounts of protein (20 µg) were resolved by the NuPAGE 4% to 20% Tris-glycine gradient gel (Invitrogen), transferred to polyvinylidene membranes, and blocked in 5% nonfat milk in phosphate-buffered saline/Tween-20. Membranes were incubated with the following antibodies: Sox2 (Cell Signaling, Danvers, MA) and α-Tubulin (Sigma-Aldrich). The membranes were developed according to the protocol for enhanced chemiluminiscence from Amersham (Piscataway, NJ).

### Neurosphere Size Analysis

To assess neurosphere size, cells derived from the dissociation of clonal single neurospheres were seeded in 96-well plates, and the size of the generated secondary spheres was assessed after 10 days. We counted 20 neurospheres per sample, and the median neurosphere size of each sample was plotted with 95% confidence intervals. Images were captured and measured using a deconvolution microscope (Zeiss, Germany).

### In Vitro Migration and Invasion Assays

For transwell migration assays, 1×10^4^ cells were plated in the top chamber with the non-coated membrane (24-well insert; pore size, 8 µm; BD Biosciences). For invasion assays, 1×10^5^ cells were plated in the top chamber with a Matrigel-coated membrane (24-well insert; pore size, 8 µm; BD Biosciences). In both assays, cells were plated in medium without serum or growth factors, and medium supplemented with 2% serum was used as a chemoattractant in the lower chamber. The cells were then incubated for 24 h. Cells that did not migrate or invade through the pores were removed by a cotton swab. Cells on the lower surface of the membrane were stained with crystal violet and counted.

### Statistical Analysis

For the in vitro experiments, statistical analyses were performed using a two-tailed Student's t test. Data are expressed as mean ± standard deviation.

## Supporting Information

Figure S1 **Molecular analysis of Sox2 in GBM specimens and cell lines.** **A.** Summary of the Sox2 staining for the 30 GBM samples presented in Fig. 1A. Each score represents the indicated range of positive percentage of intense nuclear Sox2-positive cells. Tumors were distributed according to this score analyzed independently in the central areas of the tumor or in the normal/tumoral interface. **B.** Expression of Sox2 mRNA in established glioma cell lines (n = 10) and BTSC lines (n = 12). RNA was extracted and Q-RT-PCR was performed using Sox2 expression in normal human astrocytes (NHAs; Clonetics/BioWhittaker, Walkersville, MD, USA) to normalize the data. **C.** Expression of Sox2 in BTSC lines (n = 7) and established glioma cell lines (n = 8). Normal human astrocytes (NHA) were used as a negative control and the NTERA-2 (NTs) cell line was used as a positive control. **D.** Amplification of Sox2 in 7 BTSCs and 7 established glioma cell lines. N (Normal) = normal human astrocytes; A (Amplifly) = leukemia cell line TF1, which is known to have an amplification of SOX2 gene. We used the comparative ddCt method with SYBR for Q-PCR. A primer set for the EOMES and SLITRK3 genes was used for normalization. Red dashed lines represent the normal threshold.
(TIF)

Figure S2 **Analysis of the** ***Sox2*** **promoter methylation profile in BTSCs and established cell lines. A.** Analysis of the *Sox2* gene CpG content. The positions of the CpG islands are shown. The vertical tic marks depicts the CpG's in the island. **B.** MSP analysis of the promoter CpG islands of *Sox2* in 10 BTSCs lines, 9 established glioma cell lines and normal human astrocytes (NHA). PCR products recognizing unmethylated (U) and methylated (M) CpG sites are analyzed on 2% agarose gels. C+ = positive control; *in-vitro* methylated control, C− = negative control; DNA from normal brain and ddH_2_O = water control containing no DNA.
(TIF)

Figure S3 **Role of Sox2 in self-renewal properties of GBM.** **A.** Quantification of Sox2 self-renewal capabilities of BTSCs. using a limiting dilution assay as described previously (2, 21). After primary sphere formation was noted, NSC23 cultures were dissociated and plated in 96-well microwell plates and cells were transfected with mock, vehicle (Veh), siRNA scramble (siScrbl) or two different siRNAs against Sox2, Sox2 (I) and Sox2 (II).. Final cell dilutions ranged from 200 cells/well to 1 cell/well. Cultures were fed every 2 days until day 7, when the percentage of wells not containing spheres for each cell plating density was calculated and plotted against the number of cells/well. Regression lines were plotted, and x-intercept values were calculated; these represent the number of cells required to form at least one tumor sphere in every well. **B.** Evaluation of cell cycle in Sox2-silenced BTSCs. Cells were transfected as indicated and 48 h later fixed in cold 70% ethanol (in PBS) for 20 min. Then, cells were stained with a mixture of propidium iodide (40 µg/ml) and ribonuclease A (10 mg/ml) in PBS and incubated for 30 min at 37°C and subjected to flow cytometry analysis. The data represent the means and 95% CIs of three different experiments. **C.** Expression of different proliferation markers by Q-RT-PCR in Sox2-silenced BTSCs. Sox2 was silenced as described above, and 48 h later RNA was Q-PCR analysis was performed. Quantification of the expression of the indicated genes was performed using TaqMan gene expression assays (Applied Biosystems) specific for each gene. *GAPDH* was used as an internal control. To determine relative gene expression, we used the comparative threshold cycle method.
(TIF)

Figure S4 **Role of Sox2 in the maintenance of self-renewal properties of the BTSC line NSC11.** **A.** Silencing of Sox2 in NSC11 BTSCs. Cells were transfected with two different siRNAs against Sox2 and subjected to Western blotting 48 h later. U-87 MG and the NTs cells were used as negative and positive controls, respectively. **B.** Role of Sox2 in the self-renewal capabilities of NSC11cells. NSC11 cells derived from the dissociation of clonal single neurospheres were seeded in 96-well plates, and the number of generated secondary spheres was assessed after 8–10 days. To avoid including the colonies that may have been formed by transient amplification of cells in these cultures, we counted only secondary spheres that exceeded 120 µm in diameter. To confirm that the latter were, indeed, formed by stem cells, we randomly selected at least 15 individual secondary spheres and subjected them to further, long-term (2 months) propagation in each subcloning experiment. **C.** Role of Sox2 in proliferation of NSC11 cells Cell proliferation was evaluated using the BrdU cell proliferation enzyme-linked immunosorbent assay from Calbiochem (EMD Chemicals) according to the manufacturer's recommendations. **D.** Impact of Sox2 on NSC11 sphere size. Sox2 was silenced as described above and cells derived from the dissociation of clonal single neurospheres were seeded in 96-well plates, and the size of generated secondary spheres was assessed after 10 days. Images were captured and measured using a deconvolution microscope (Zeiss). We counted 20 neurospheres per sample and means with 95% CIs were plotted.
(TIF)

Figure S5 **Evaluation of the role of Sox2 on the established glioma cell lines.** **A.** Overexpression of Sox2 in Sox2-negative glioma cell lines. U-87 MG cells were left untreated (mock) or infected (100 MOIs) with AdLacZ or AdSox2. Sox2 expression levels were evaluated at 24, 48, and 72 h using Western blotting. The NTs cell line was used as a positive control. **B.** Assessment of self-renewal in U-87 MG cells expressing exogenous Sox2. U-87 MG cells were left untreated (mock) or infected (at 100 MOIs) with an empty adenoviral vector (AdCMV), or AdLacZ or AdSox2. The U-87 MG cell line was cultured using the conditions described above for neurosphere cultures and allowed to grow for 48 h. Cells were then seeded in 96-well plates, and the number of generated secondary spheres was assessed after 8–10 days. **C and D.** Cell size evaluation in the Sox2-overexpressing U-87 MG cell line. U-87 MG cells were cultured in neurosphere medium and allow to form neurospheres. Cells derived from the dissociation of clonal single neurospheres were seeded in 96-well plates, and the size of the generated secondary spheres was assessed after 10 days. Images were captured and measured using a deconvolution microscope (Zeiss). We counted 20 neurospheres per sample and the means with 95% CIs were plotted. **E.** Cell viability analysis in the Sox2-overexpressing U-87 MG cell line. U-87 MG cells were seeded then treated with the indicated treatments. Twenty-four hours later, cells were tripsinized and seeded at 2000 cells/well in 96-well plates and allowed to grow for 7 days. MTT experiments were performed to quantify cell viability. **F.** Cell proliferation analysis in the Sox2-overexpressing U-87 MG cell line. Cell proliferation was evaluated using the BrdU cell proliferation enzyme-linked immunosorbent assay from Calbiochem (EMD Chemicals, Gibbstown, NJ) according to the manufacturer's recommendations. U-87 MG cells were treated as above **G.** Assessment of self-renewal in Sox2-positive glioma cell lines U373 and LN229. Both cell lines were allowed to grow in either 10% serum or conditions to culture neurospheres. Cells were then seeded in 96-well plates, and the number of generated spheres was assessed after 10 days. Results of clones growing as attached cultures were quantified (please note that no neuropsheres were observed). Data are expressed as the means with 95% CIs from three independent experiments.
(TIF)

Figure S6 **Role of Sox2 in the invasive phenotype of gliomas.** **A.** Assessment of invasion in Sox2-silenced BTSCs (NSC11, NSC7-26) and Sox2-positive glioma (LN229) cell lines. For transwell invasion assays NSC11, NSC6-27, or LN229 cells were left untreated or transfected with a scramble siRNA (50 nM), Sox2 (I), or Sox2(II) (50 nM; Ambion). Twenty-four h later, 1×10^5^ cells were plated in the top chamber with a Matrigel-coated membrane (24-well insert; pore size, 8 µm; BD Biosciences). Cells were plated in medium without serum or growth factors, and medium supplemented with 2% serum was used as a chemoattractant in the lower chamber. The cells were then incubated for 24 h. Cells that did not invade through the pores were removed using a cotton swab. Cells on the lower surface of the membrane were stained with crystal violet and counted. Quantification of the invasion is expressed as the number of invasive cells per field. Bottom, representative micrographs of the transwell invasion assay (10× magnification). **B.** Analysis of invasion markers in Sox2-silenced NSC11, NSC7-26 and LN229 cells. Sox2 was silenced as described above. Quantification of the expression of the indicated genes was performed using TaqMan gene expression assays (Applied Biosystems) specific for each gene. *GAPDH* was used as an internal control. For normalization, the cDNA equivalent to input RNA was measured in duplicate for GAPDH transcripts by RT-PCR. To determine relative gene expression, we used the comparative threshold cycle method. **C.** Assessment of migration in Sox2-overexpressing glioma cell lines by transwell migration. U-87 MG cells were left untreated or infected with 100 MOIs of AdCMV, AdLacZ cDNA, or AdSox2. Twenty-four h later, 1×10^4^ cells were plated in the top chamber with a noncoated membrane (24-well insert; pore size, 8 µm; BD Biosciences). Cells were plated in medium without serum or growth factors, and medium supplemented with 2% serum was used as a chemoattractant in the lower chamber. The cells were then incubated for 24 h. Cells that did not invade through the pores were removed using a cotton swab. Cells on the lower surface of the membrane were stained with crystal violet and counted. Top, quantification of the invasion expressed as the number of migrating cells per HPF. Bottom, representative micrographs of the transwell migration assay (10× magnification).
(TIF)

Text S1 **Primer sequences.**
(DOC)
