# Expression of concern: Genetic and epigenetic modifications of Sox2 contribute to the invasive phenotype of malignant gliomas

**DOI:** 10.1371/journal.pone.0355894

**Published:** 2026-08-12

**Authors:** The PLOS One Editors

After this article [1] was published, co-corresponding author MMA notified PLOS of an error in Fig 5, specifically that the siScrbl panels in Figs 5A and 5B overlap with each other.

Further editorial review noted additional concerns in S2 and S5 Figs. Specifically;

- When flipped horizontally, the Sox2 (MSP) Cell lines panels in Fig S2B appear similar to each other.
- Regions within the AdLacZ panel of Fig S5C appear similar to each other.

The author stated that the siScrbl panel in Fig 5B and the lower Sox2 (MSP) cell line panel in Fig S2B are incorrect and provided a corrected lower Sox2 (MSP) cell line panel from the original experiments, provided here as S1 File with the original images underlying both cell lines panels in Fig S2B. They also provided a corrected siScrbl panel for Fig 5B but stated that the original images underlying the siSox2 panels in Figs 5A and 5B could not be located. The underlying image provided in support of the siScrbl panel of Fig 5A does not appear to match the corresponding panel in [1]. The corrected Fig 5B siScrbl panel from the original experiments has been included with this notice as S2 File; however, the underlying image for this panel was not provided. The author provided the original images underlying the Mock, CMV, and AdLacZ panels in Fig S5C (included here as S3 File).

The *PLOS One* Editors issue this Expression of Concern to notify readers of the above concerns and to relay the underlying data provided.

## Supporting information

S1 File Corrected lower Sox2 (MSP) cell lines panel in Fig S2B, along with the original images underlying both Cell lines panels. (ZIP)

S2 File Corrected Fig 5B siScrbl panel. (ZIP)

S3 File Original images underlying Mock, CMV, and AdLacZ panels in Fig S5C. (ZIP)
