# Supplementary figures and images for: Genetic and Epigenetic Modifications of Sox2 Contribute to the Invasive Phenotype of Malignant Gliomas
Source: PLoS One. 2011 Nov 1;6(11):e26740. doi: 10.1371/journal.pone.0026740 (PMC3206066; doi:10.1371/journal.pone.0026740)

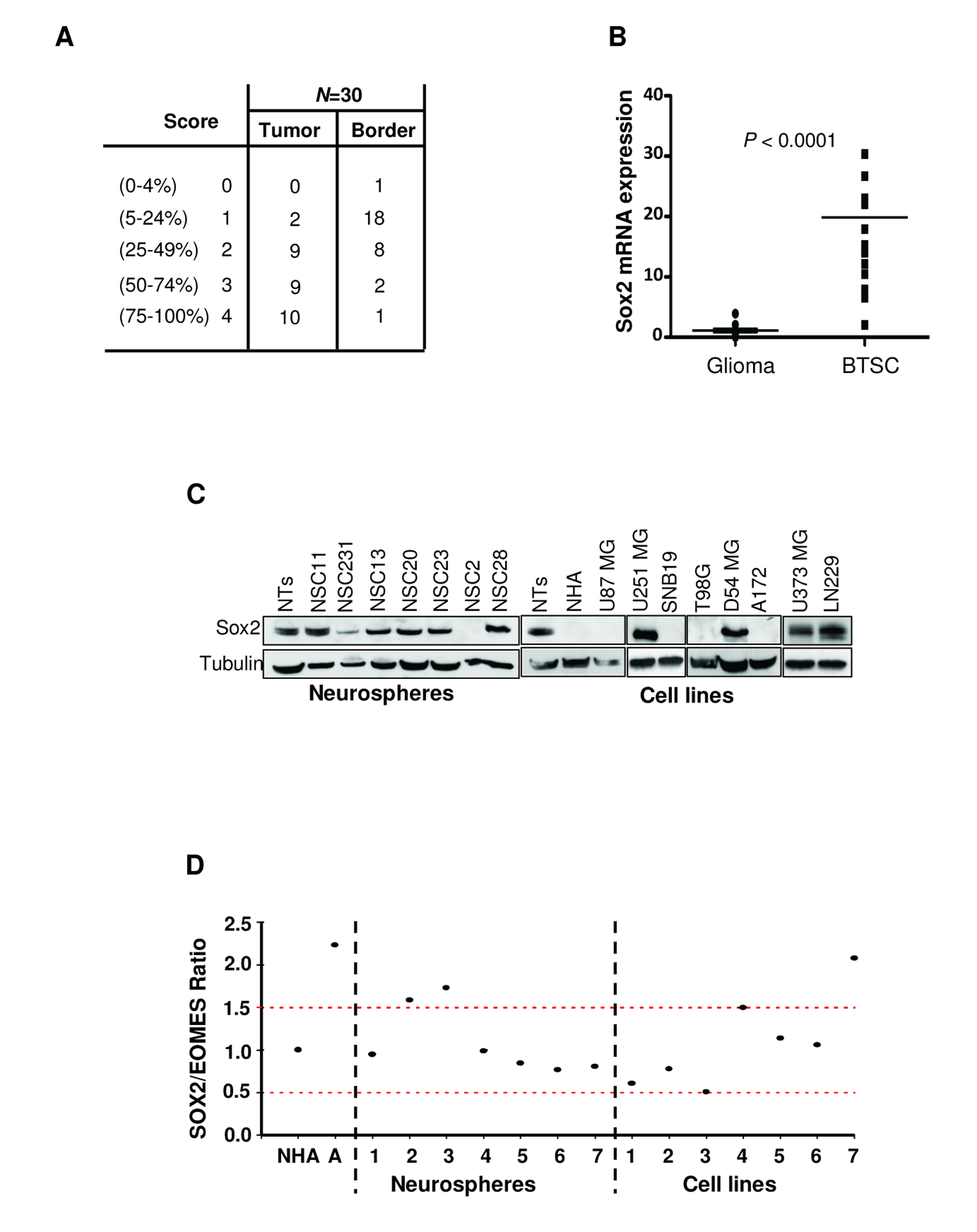

Supplement: Figure S1 — Molecular analysis of Sox2 in GBM specimens and cell lines. A. Summary of the Sox2 staining for the 30 GBM samples presented in Fig. 1A. Each score represents the indicated range of positive percentage of intense nuclear Sox2-positive cells. Tumors were distributed according to this score analyzed independently in the central areas of the tumor or in the normal/tumoral interface. B. Expression of Sox2 mRNA in established glioma cell lines (n = 10) and BTSC lines (n = 12). RNA was extracted and Q-RT-PCR was performed using Sox2 expression in normal human astrocytes (NHAs; Clonetics/BioWhittaker, Walkersville, MD, USA) to normalize the data. C. Expression of Sox2 in BTSC lines (n = 7) and established glioma cell lines (n = 8). Normal human astrocytes (NHA) were used as a negative control and the NTERA-2 (NTs) cell line was used as a positive control. D. Amplification of Sox2 in 7 BTSCs and 7 established glioma cell lines. N (Normal) = normal human astrocytes; A (Amplifly) = leukemia cell line TF1, which is known to have an amplification of SOX2 gene. We used the comparative ddCt method with SYBR for Q-PCR. A primer set for the EOMES and SLITRK3 genes was used for normalization. Red dashed lines represent the normal threshold. (TIF) [file pone.0026740.s001.tif]

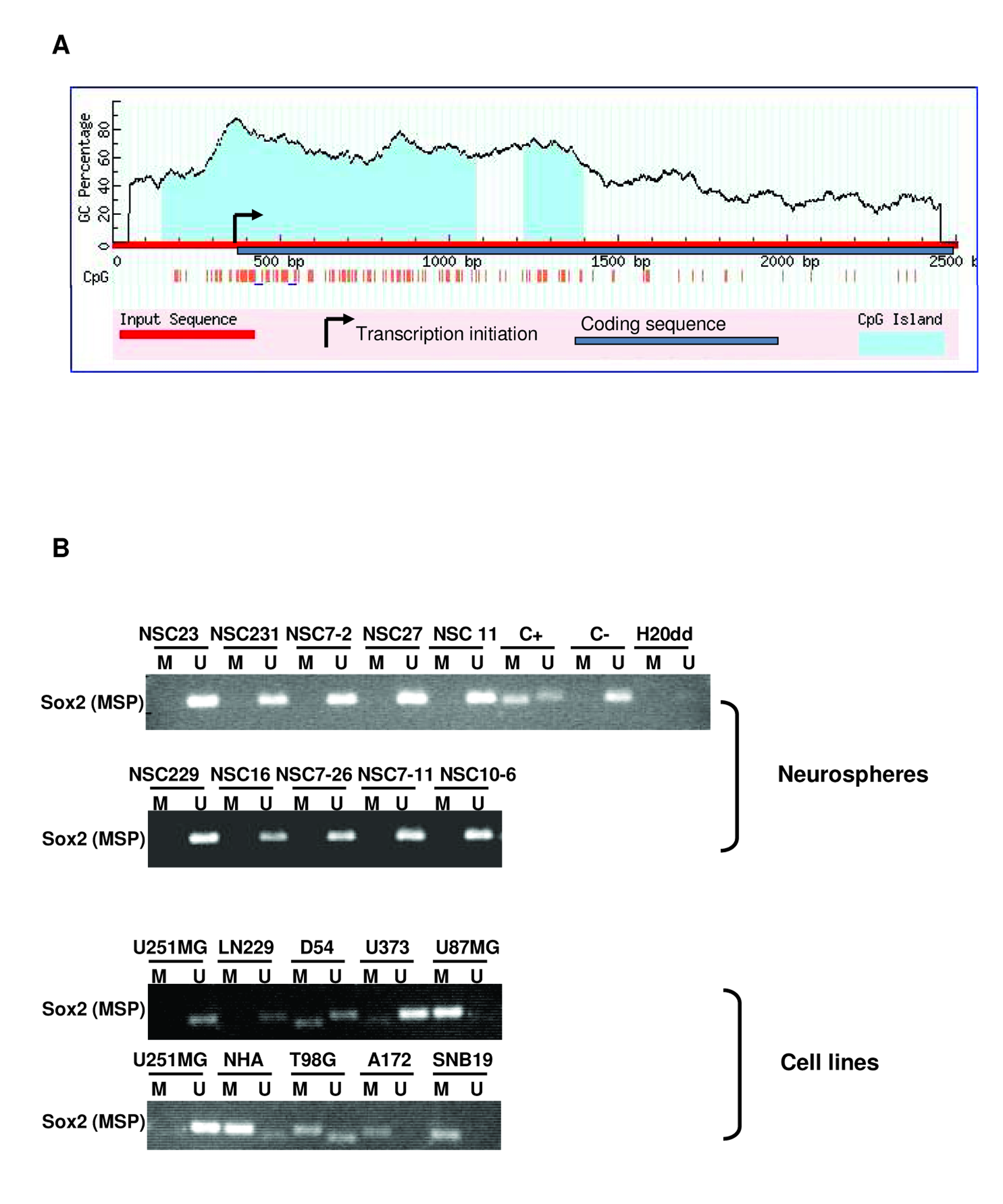

Supplement: Figure S2 — Analysis of the Sox2 promoter methylation profile in BTSCs and established cell lines. A. Analysis of the Sox2 gene CpG content. The positions of the CpG islands are shown. The vertical tic marks depicts the CpG's in the island. B. MSP analysis of the promoter CpG islands of Sox2 in 10 BTSCs lines, 9 established glioma cell lines and normal human astrocytes (NHA). PCR products recognizing unmethylated (U) and methylated (M) CpG sites are analyzed on 2% agarose gels. C+ = positive control; in-vitro methylated control, C− = negative control; DNA from normal brain and ddH2O = water control containing no DNA. (TIF) [file pone.0026740.s002.tif]

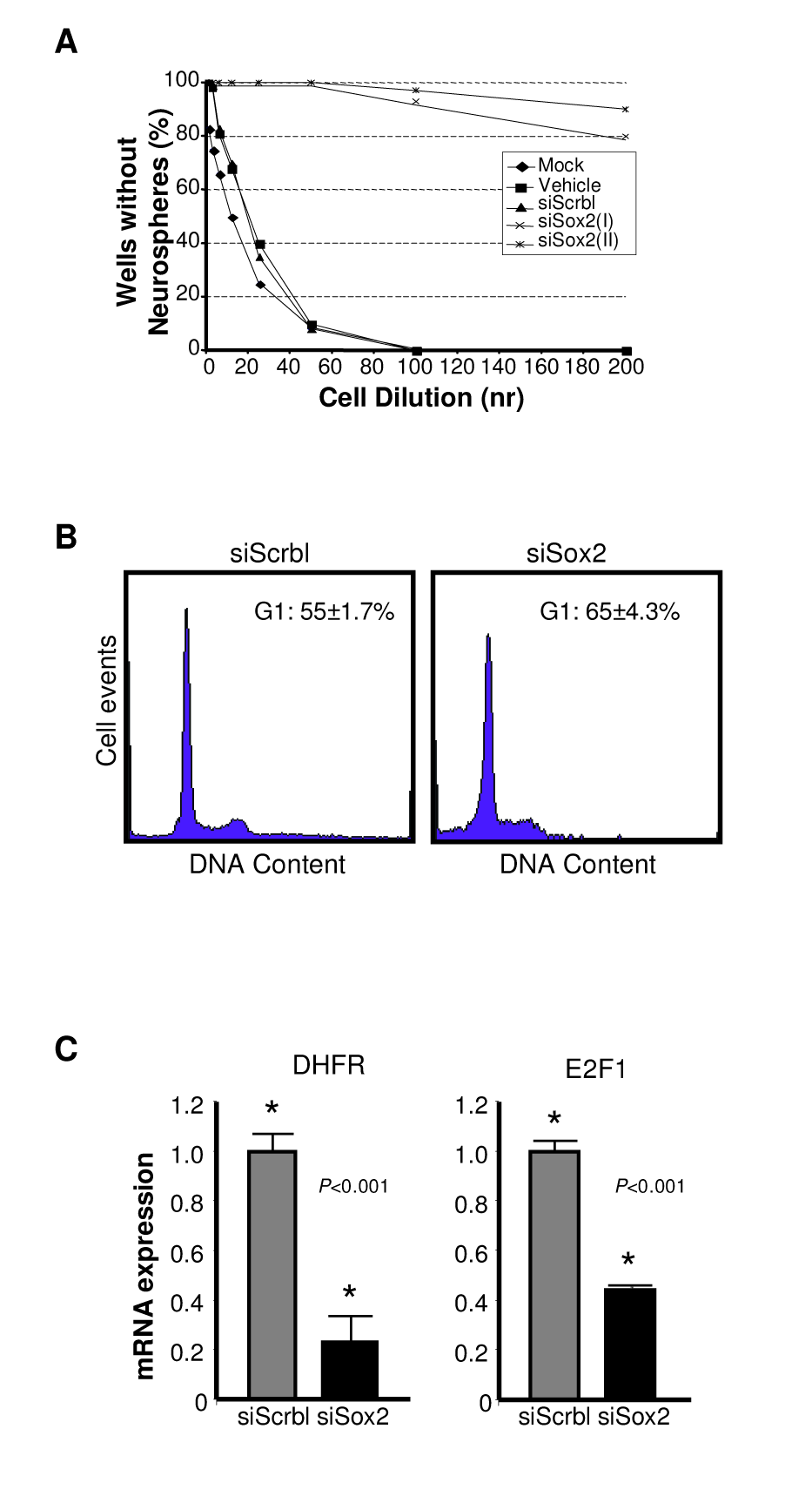

Supplement: Figure S3 — Role of Sox2 in self-renewal properties of GBM. A. Quantification of Sox2 self-renewal capabilities of BTSCs. using a limiting dilution assay as described previously (2, 21). After primary sphere formation was noted, NSC23 cultures were dissociated and plated in 96-well microwell plates and cells were transfected with mock, vehicle (Veh), siRNA scramble (siScrbl) or two different siRNAs against Sox2, Sox2 (I) and Sox2 (II).. Final cell dilutions ranged from 200 cells/well to 1 cell/well. Cultures were fed every 2 days until day 7, when the percentage of wells not containing spheres for each cell plating density was calculated and plotted against the number of cells/well. Regression lines were plotted, and x-intercept values were calculated; these represent the number of cells required to form at least one tumor sphere in every well. B. Evaluation of cell cycle in Sox2-silenced BTSCs. Cells were transfected as indicated and 48 h later fixed in cold 70% ethanol (in PBS) for 20 min. Then, cells were stained with a mixture of propidium iodide (40 µg/ml) and ribonuclease A (10 mg/ml) in PBS and incubated for 30 min at 37°C and subjected to flow cytometry analysis. The data represent the means and 95% CIs of three different experiments. C. Expression of different proliferation markers by Q-RT-PCR in Sox2-silenced BTSCs. Sox2 was silenced as described above, and 48 h later RNA was Q-PCR analysis was performed. Quantification of the expression of the indicated genes was performed using TaqMan gene expression assays (Applied Biosystems) specific for each gene. GAPDH was used as an internal control. To determine relative gene expression, we used the comparative threshold cycle method. (TIF) [file pone.0026740.s003.tif]

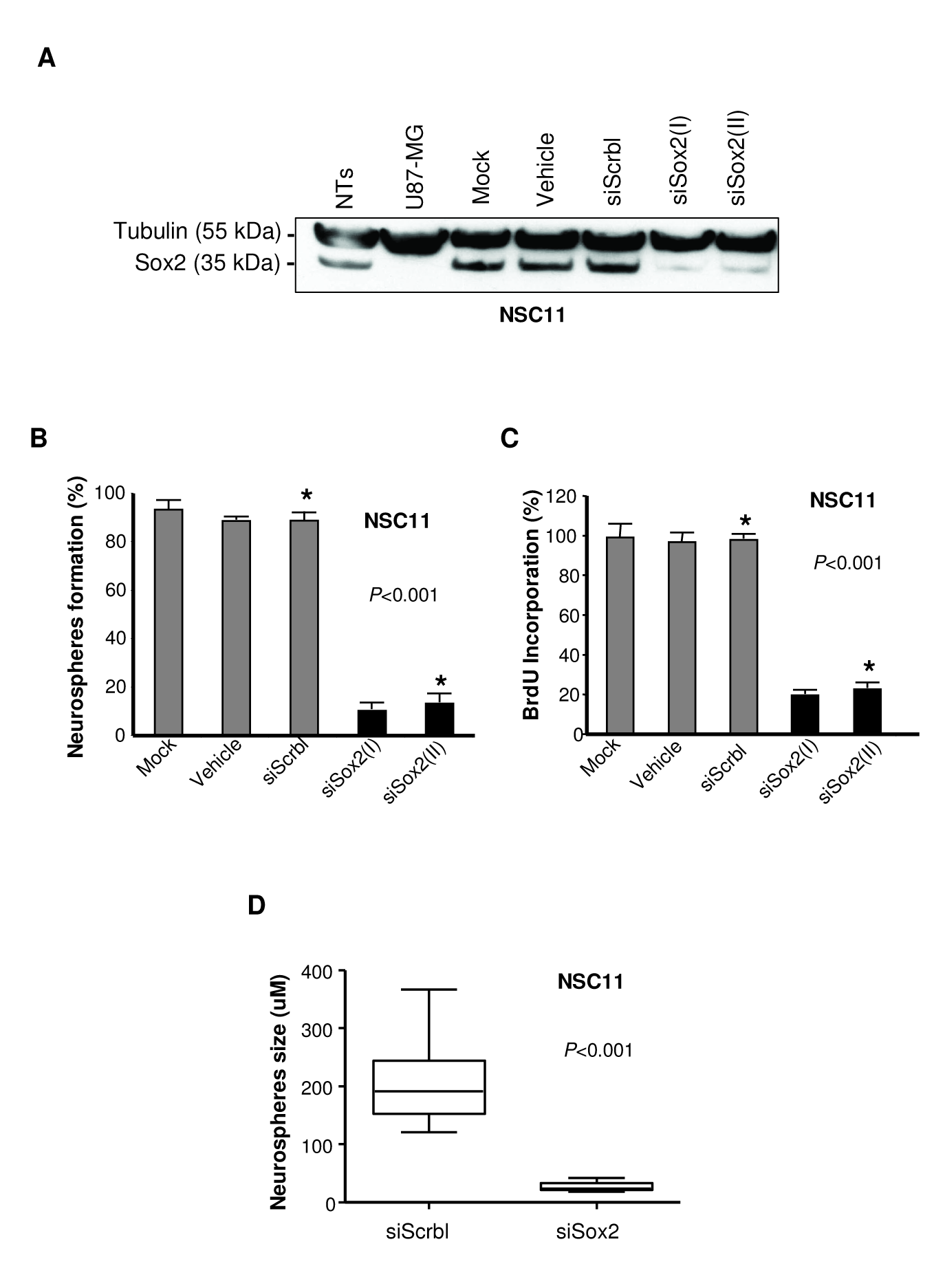

Supplement: Figure S4 — Role of Sox2 in the maintenance of self-renewal properties of the BTSC line NSC11. A. Silencing of Sox2 in NSC11 BTSCs. Cells were transfected with two different siRNAs against Sox2 and subjected to Western blotting 48 h later. U-87 MG and the NTs cells were used as negative and positive controls, respectively. B. Role of Sox2 in the self-renewal capabilities of NSC11cells. NSC11 cells derived from the dissociation of clonal single neurospheres were seeded in 96-well plates, and the number of generated secondary spheres was assessed after 8–10 days. To avoid including the colonies that may have been formed by transient amplification of cells in these cultures, we counted only secondary spheres that exceeded 120 µm in diameter. To confirm that the latter were, indeed, formed by stem cells, we randomly selected at least 15 individual secondary spheres and subjected them to further, long-term (2 months) propagation in each subcloning experiment. C. Role of Sox2 in proliferation of NSC11 cells Cell proliferation was evaluated using the BrdU cell proliferation enzyme-linked immunosorbent assay from Calbiochem (EMD Chemicals) according to the manufacturer's recommendations. D. Impact of Sox2 on NSC11 sphere size. Sox2 was silenced as described above and cells derived from the dissociation of clonal single neurospheres were seeded in 96-well plates, and the size of generated secondary spheres was assessed after 10 days. Images were captured and measured using a deconvolution microscope (Zeiss). We counted 20 neurospheres per sample and means with 95% CIs were plotted. (TIF) [file pone.0026740.s004.tif]

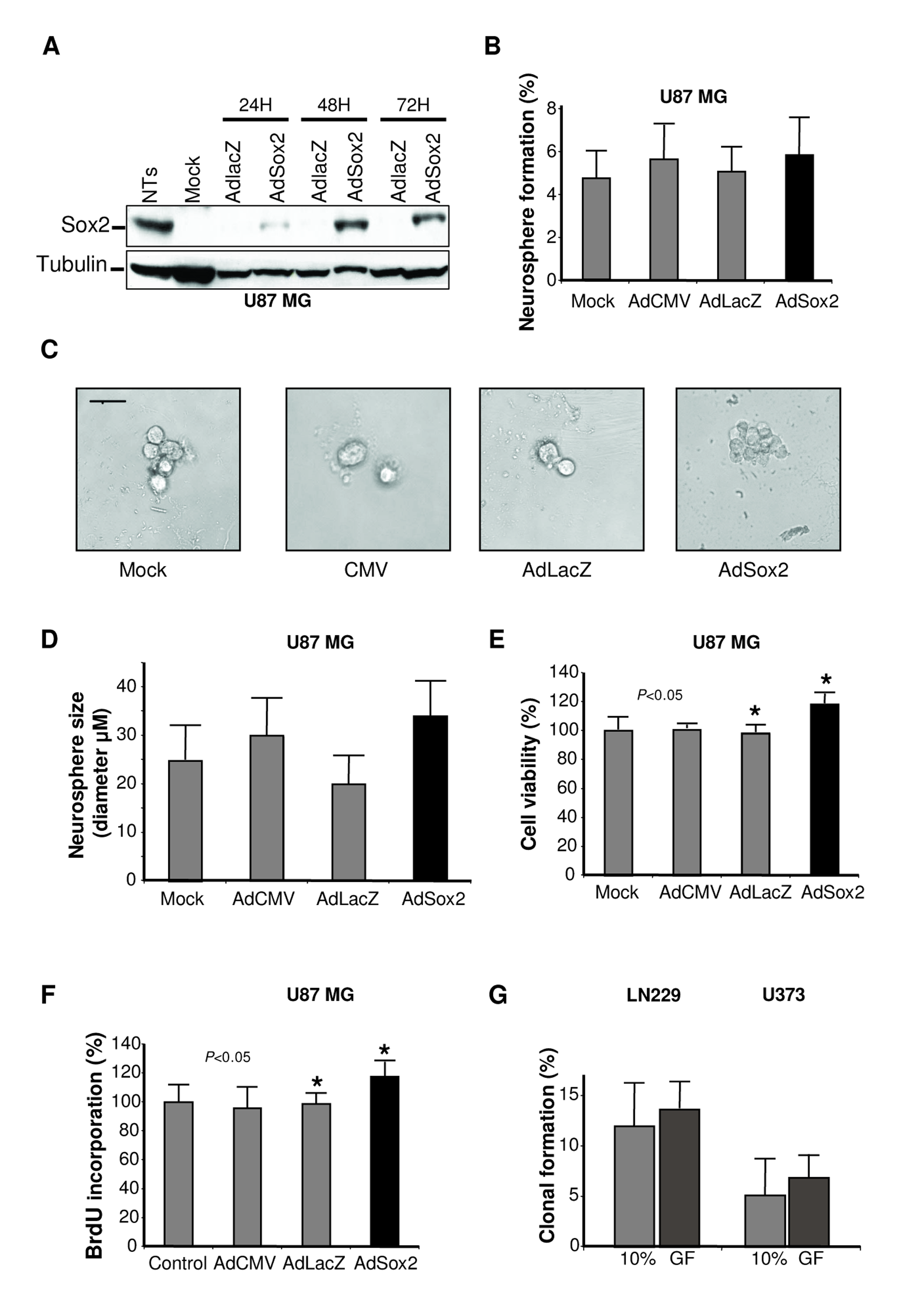

Supplement: Figure S5 — Evaluation of the role of Sox2 on the established glioma cell lines. A. Overexpression of Sox2 in Sox2-negative glioma cell lines. U-87 MG cells were left untreated (mock) or infected (100 MOIs) with AdLacZ or AdSox2. Sox2 expression levels were evaluated at 24, 48, and 72 h using Western blotting. The NTs cell line was used as a positive control. B. Assessment of self-renewal in U-87 MG cells expressing exogenous Sox2. U-87 MG cells were left untreated (mock) or infected (at 100 MOIs) with an empty adenoviral vector (AdCMV), or AdLacZ or AdSox2. The U-87 MG cell line was cultured using the conditions described above for neurosphere cultures and allowed to grow for 48 h. Cells were then seeded in 96-well plates, and the number of generated secondary spheres was assessed after 8–10 days. C and D. Cell size evaluation in the Sox2-overexpressing U-87 MG cell line. U-87 MG cells were cultured in neurosphere medium and allow to form neurospheres. Cells derived from the dissociation of clonal single neurospheres were seeded in 96-well plates, and the size of the generated secondary spheres was assessed after 10 days. Images were captured and measured using a deconvolution microscope (Zeiss). We counted 20 neurospheres per sample and the means with 95% CIs were plotted. E. Cell viability analysis in the Sox2-overexpressing U-87 MG cell line. U-87 MG cells were seeded then treated with the indicated treatments. Twenty-four hours later, cells were tripsinized and seeded at 2000 cells/well in 96-well plates and allowed to grow for 7 days. MTT experiments were performed to quantify cell viability. F. Cell proliferation analysis in the Sox2-overexpressing U-87 MG cell line. Cell proliferation was evaluated using the BrdU cell proliferation enzyme-linked immunosorbent assay from Calbiochem (EMD Chemicals, Gibbstown, NJ) according to the manufacturer's recommendations. U-87 MG cells were treated as above G. Assessment of self-renewal in Sox2-positive glioma cell lines U373 and LN [file pone.0026740.s005.tif]

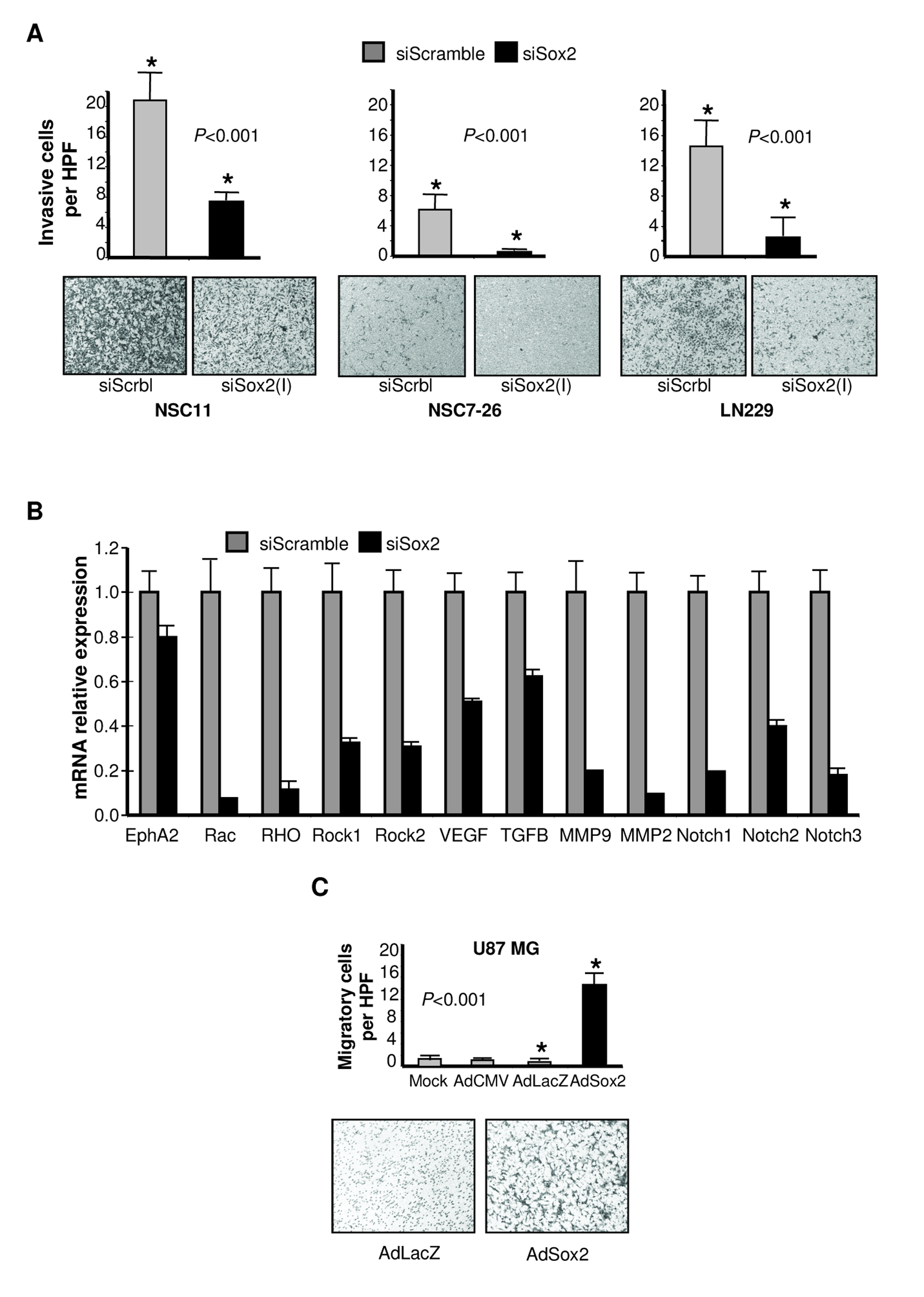

Supplement: Figure S6 — Role of Sox2 in the invasive phenotype of gliomas. A. Assessment of invasion in Sox2-silenced BTSCs (NSC11, NSC7-26) and Sox2-positive glioma (LN229) cell lines. For transwell invasion assays NSC11, NSC6-27, or LN229 cells were left untreated or transfected with a scramble siRNA (50 nM), Sox2 (I), or Sox2(II) (50 nM; Ambion). Twenty-four h later, 1×10^5^ cells were plated in the top chamber with a Matrigel-coated membrane (24-well insert; pore size, 8 µm; BD Biosciences). Cells were plated in medium without serum or growth factors, and medium supplemented with 2% serum was used as a chemoattractant in the lower chamber. The cells were then incubated for 24 h. Cells that did not invade through the pores were removed using a cotton swab. Cells on the lower surface of the membrane were stained with crystal violet and counted. Quantification of the invasion is expressed as the number of invasive cells per field. Bottom, representative micrographs of the transwell invasion assay (10× magnification). B. Analysis of invasion markers in Sox2-silenced NSC11, NSC7-26 and LN229 cells. Sox2 was silenced as described above. Quantification of the expression of the indicated genes was performed using TaqMan gene expression assays (Applied Biosystems) specific for each gene. GAPDH was used as an internal control. For normalization, the cDNA equivalent to input RNA was measured in duplicate for GAPDH transcripts by RT-PCR. To determine relative gene expression, we used the comparative threshold cycle method. C. Assessment of migration in Sox2-overexpressing glioma cell lines by transwell migration. U-87 MG cells were left untreated or infected with 100 MOIs of AdCMV, AdLacZ cDNA, or AdSox2. Twenty-four h later, 1×10^4^ cells were plated in the top chamber with a noncoated membrane (24-well insert; pore size, 8 µm; BD Biosciences). Cells were plated in medium without serum or growth factors, and medium supplemented with 2% serum was used as a chemoattractant in the lower chamber. The cel [file pone.0026740.s006.tif]
