# Supplementary material for: Genetic and Epigenetic Modifications of Sox2 Contribute to the Invasive Phenotype of Malignant Gliomas
Source: PLoS One. 2011 Nov 1;6(11):e26740. doi: 10.1371/journal.pone.0026740 (PMC3206066; doi:10.1371/journal.pone.0026740)
Supplement: Text S1 — Primer sequences. (DOC) [file pone.0026740.s007.doc]

**Primer sequences:**

|  | **Forward** | **Reverse** |
| --- | --- | --- |
| **GAPDH** | ATGGGGAAGGTGAAGGTCGG | GACGGTGCCATGGAATTTGC |
| **SOX2** | TACAGCATGTCCTACTCGCAG | GAGGAAGAGGTAACCACAGGG |
| **NESTIN** | AGACTTCCCTCAGTTTAGG | CAGGTGTCTCAAGGGTAGCAG |
| **GFAP** | GCAGAGATGATGGAGCTCAATGACC | GTTTCATCCTGGAGCTTCTGCCTCA |
| **BMP4** | cttcagtctggggaggag | gatgaggtgcccaggcaC |
| **B-Tubulin III** | GCGAGATGTACGAAGACGAC | TTTAGACACTGCTGGCTTCG |
| **OCT4** | TTCAGCCAAACGACCATC | CAGGTTGCCTCTCACTCG |
| **REST** | AGCGAGTACCACTGGAGGAA | CTGAATGAGTCCGCATGTGT |
| **S-Meth1** | CGtATGGAtAGTTACGCGtAtATGAAC | GTaCGCATTaAaaCCCGAaTaCTaCG |
| **S-UnM1** | GAAttAGtGtATGGAtAGTTAtGtGtAtATGAAtG | CTaCaCCaTaCaCATTaAaaCCCaaaTaCTaCa |
| **S-Meth2** | GGtCGGGttCGCGtAtAGC | CCGCCGCGaTaaAaTTaCCG |
| **S-UnM2** | GtGGGttGGGtttGtGtAtAGtG | CCaaCCaCCaCCaCCaTaaAaTTaCCa |
